# Expression of HPV16 E5 down-modulates the TGFbeta signaling pathway

**DOI:** 10.1186/1476-4598-12-38

**Published:** 2013-05-07

**Authors:** Deborah French, Francesca Belleudi, Maria Vittoria Mauro, Francesca Mazzetta, Salvatore Raffa, Vincenza Fabiano, Antonio Frega, Maria Rosaria Torrisi

**Affiliations:** 1 Istitute Pasteur-Fondazione Cenci Bolognetti, Department of Clinical and Molecular Medicine, Sapienza University of Roma, Rome, Italy; 2 S. Andrea Hospital, Rome, Italy

**Keywords:** Human papillomavirus, HPV16 E5, TGFβ signaling, TGFβRII, SMAD

## Abstract

**Background:**

Infection with high-risk human papillomavirus (HR-HPV) genotypes, mainly HPV16 and HPV18, is a major risk factor for cervical cancer and responsible for its progression. While the transforming role of the HPV E6 and E7 proteins is more characterized, the molecular mechanisms of the oncogenic activity of the E5 product are still only partially understood, but appear to involve deregulation of growth factor receptor expression. Since the signaling of the transforming growth factor beta (TGFbeta) is known to play crucial roles in the epithelial carcinogenesis, aim of this study was to investigate if HPV16 E5 would modulate the TGF-BRII expression and TGFbeta/Smad signaling.

**Findings:**

The HPV16 E5 mRNA expression pattern was variable in low-grade squamous intraepithelial lesions (LSIL), while homogeneously reduced in high-grade lesions (HSIL). Parallel analysis of TGFBRII mRNA showed that the receptor transcript levels were also variable in LSILs and inversely related to those of the viral protein. In vitro quantitation of the TGFBRII mRNA and protein in human keratinocytes expressing 16E5 in a dose-dependent and time-dependent manner showed a progressive down-modulation of the receptor. Phosphorylation of Smad2 and nuclear translocation of Smad4 were also decreased in E5-expressing cells stimulated with TGFbeta1.

**Conclusions:**

Taken together our results indicate that HPV16 E5 expression is able to attenuate the TGFbeta1/Smad signaling and propose that this loss of signal transduction, leading to destabilization of the epithelial homeostasis at very early stages of viral infection, may represent a crucial mechanism of promotion of the HPV-mediated cervical carcinogenesis.

## Introduction

The infection with high-risk human papillomavirus (HR-HPV) genotypes, particularly the HPV16 and HPV18 viruses, is a major risk factor for cervical cancer and appears to be responsible for its progression [1-3]. While the transforming role of the HPV E6 and E7 proteins is well characterized, the molecular mechanisms of the oncogenic activity of the E5 product of the virus are still only partially understood for a recent review, see [4]. HPV16 E5 expression is lost during tumor progression as a result of viral genome integration: we have recently reported that the E5 expression pattern is extremely variable in low-grade squamous intraepithelial lesions (LSIL), while it is reduced and more homogeneous in high-grade lesions (HSIL) [5]. When expressed, the E5 protein is known to play critical roles at the early stages of infection by co-operation with E6 and E7 affecting proliferation, differentiation and apoptosis [4] and these multiple functions appear to be dependent on its ability to enhance the signaling pathways of mitogenic growth factor receptors such as the epidermal growth factor receptor (EGFR) [6]. However, 16E5 protein is also capable to down-regulate the expression of receptors responsible for epithelial differentiation, such as the keratinocyte growth factor receptor (KGFR/FGFR2b), in order to perturb the physiological tissue homeostasis and cell stratification [7].

Signaling of the transforming growth factor β (TGFβ) is known to play crucial roles in the control of a number of key physiological cell processes, such as proliferation, differentiation, motility and death, and is involved in the pathogenesis of many different human diseases including cancer for a recent review, see [8]. Members of the TGFβ family bind to cell surface heterodimers composed of type I and type II serine/threonine receptors (TGFβRI and TGFβRII) and this binding triggers an intracellular signal transduction mediated by SMAD proteins and regulated by their phosphorylation and activation for a recent review, see [9]. The cellular response to TGFβ signaling is highly variable, ranging from tumor suppressive to tumor promoting functions, and depends on the cellular context and tissue microenvironment [8]. Dysregulated expression and activity of TGFβ, TGFβRI/II and SMADs have been frequently described in human cancer in association with tumor progression [8]. It has been proposed that altered expression of TGFβ might be involved in cervical carcinogenesis [10,11] and that TGFβ1 promotes chromosomal instability in HPV-infected cervical cells [12]. Interestingly, mice lacking TGFβRII develop spontaneous anal and genital squamous carcinomas [13], previewed in [14], demonstrating that the loss of TGFβ signaling promotes the carcinogenesis in those stratified epithelia, which represent the tissue targets of mucosal HPV infection. Consistent with the hypothesis that the molecular mechanisms of HPV transformation may involve the TGFβ/TGFβR axis, the E7 transforming protein of HPV16 is able to down-regulate the TGF-βRII expression and signaling in a E7 transgenic mouse model suggesting a key role of this pathway [15]. Therefore, it is possible that, in early infection and in the LSIL/HSIL context, HPV E5 might exert its oncogenic activity through modulation of the TGF-βRII expression and TGFβ signaling.

Our present study was aimed to analyze in vivo the possible HPV E5-induced alteration of the TGFβ signaling pathway in human lesions as well as its in vitro modulation under the expression of the E5 oncogenic protein. Since the local tissue concentration of the cytokine TGFβ might be extremely variable, depending on the inflammatory and immunological microenvironment [11,16], we focused both in vivo and in vitro on the expression of the receptor, since its levels are thought to strictly regulate the specificity of the TGFβ signaling and the biological activity of the cytokine [17]. In addition, because the biological behaviour of human HaCaT keratinocytes is drastically affected by TGFβ/Smad signaling [18], we took advantage of this sensitive cellular model in which 16E5 expression alone can be induced by trasfection in dose and time controlled manner.

## Findings and discussion

### Modulation of HPV16 E5 and TGFβRII in LSILs and HSILs

**Figure 1:**
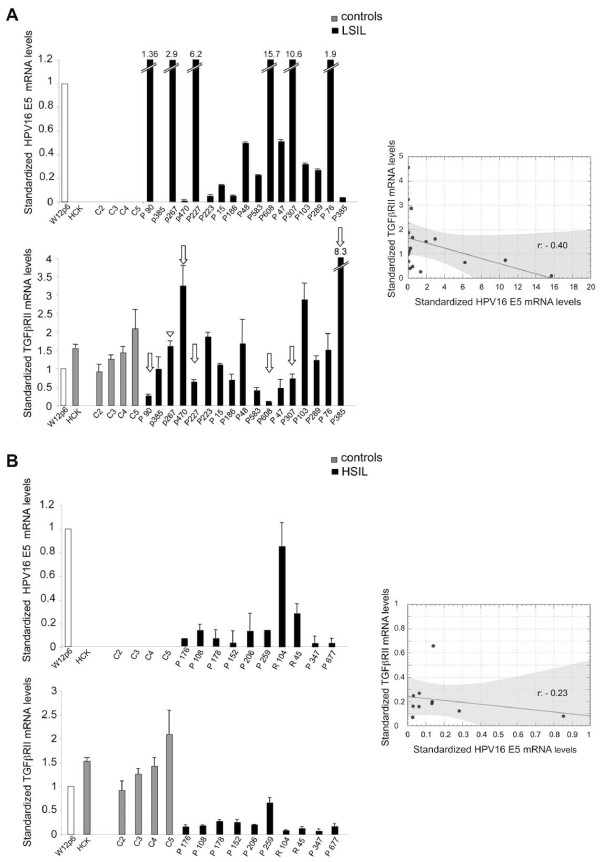
**Expression of TGFβRII and HPV16 E5 in LSILs and HSILs.** HPV16 E5 and TGFβRII mRNA levels were evaluated by real-time RT-PCR in LSILs (**A**) and in HSILs (**B**) and normalized respect to W12p6 cells. Arrows point to samples showing inverse correlation of the transcript levels. Results are expressed as mean ± 95% confidence interval (CI). Statistical analysis was performed and shown in the scatter diagrams. r: Pearson correlation coefficient values, black lines: regression lines, gray areas: areas of 95% confidence band.

In order to investigate the possible existence of a relationship between the E5 viral protein of HPV16 and the TGFβRII expression, we first quantified the 16E5 and TGFβRII mRNA levels in various samples of low grade (LSIL) and high grade (HSIL) squamous intra-epithelial lesions by real-time RT-PCR and normalized them respect to their levels in the HPV16-positive cervical epithelial cell line W12 [19] at the passage 6 (W12p6), in which ~100 to 200 copies per cell of the E5-expressing HPV episomes were retained [20]; our unpublished data. Results showed a great variability of 16E5 transcript levels in LSILs, which could be related to a high variability of episomal/integrated HPV16 distribution (Figure 1A, upper panel) as expected [5]. The primary culture of normal human ectocervical keratinocytes (HCK) as well as the negative controls (C2-C5) were all negative for 16E5 mRNA (Figure 1A, upper panel). In contrast, as previously reported [5], in all HSILs the expression of 16E5 mRNA resulted lower if compared to W12p6, presumably as a result of viral integration. The parallel analysis of TGFβRII mRNA showed that the receptor transcript levels were also highly variable in LSILs and appeared inversely linked to those of the viral protein (Figure 1A, lower panel): in fact, particularly in P90, P227 and P608 samples, high levels of 16E5 transcript corresponded to low levels of the receptor mRNA, while in p470 and P385 samples very low levels of the viral protein transcript corresponded to high levels of TGFβRII mRNA (Figure 1A, lower panel, arrows). Only in a single sample (p267) with high levels of 16E5 mRNA the amount of TGFβRII appeared not modulated and comparable to that of control samples (Figure 1A, lower panel, arrowhead). In contrast, a significant homogeneous down-modulation of TGFβRII mRNA expression compared to either the controls and the W12p6 cells (p < 0.01 vs controls) was found in HSILs (Figure 1B, lower panel), suggesting here the possible independence of the receptor modulation from 16E5 expression when viral integration or mixed episomal/integrated states are expected with more frequency (Figure 1B upper panel) and consistent with the proposed down-modulating effect of the HPV E7 protein on TGFβRII in the mouse model [15]. Results are expressed as mean ± 95% confidence interval (CI). Statistical analysis was performed as reported in the Additional file 1 Materials and Methods. Therefore, while in early steps of HPV infection the expression of TGFβRII can be regulated by the E5 protein, in more advanced lesions, characterized by frequent viral integration and loss of E5 expression, the receptor transcription would be stably controlled by other HPV transforming proteins, such as the E7 oncogene product.

### 16E5 expression induces TGFβRII down-modulation and attenuates TGFβ/Smad signaling

**Figure 2:**
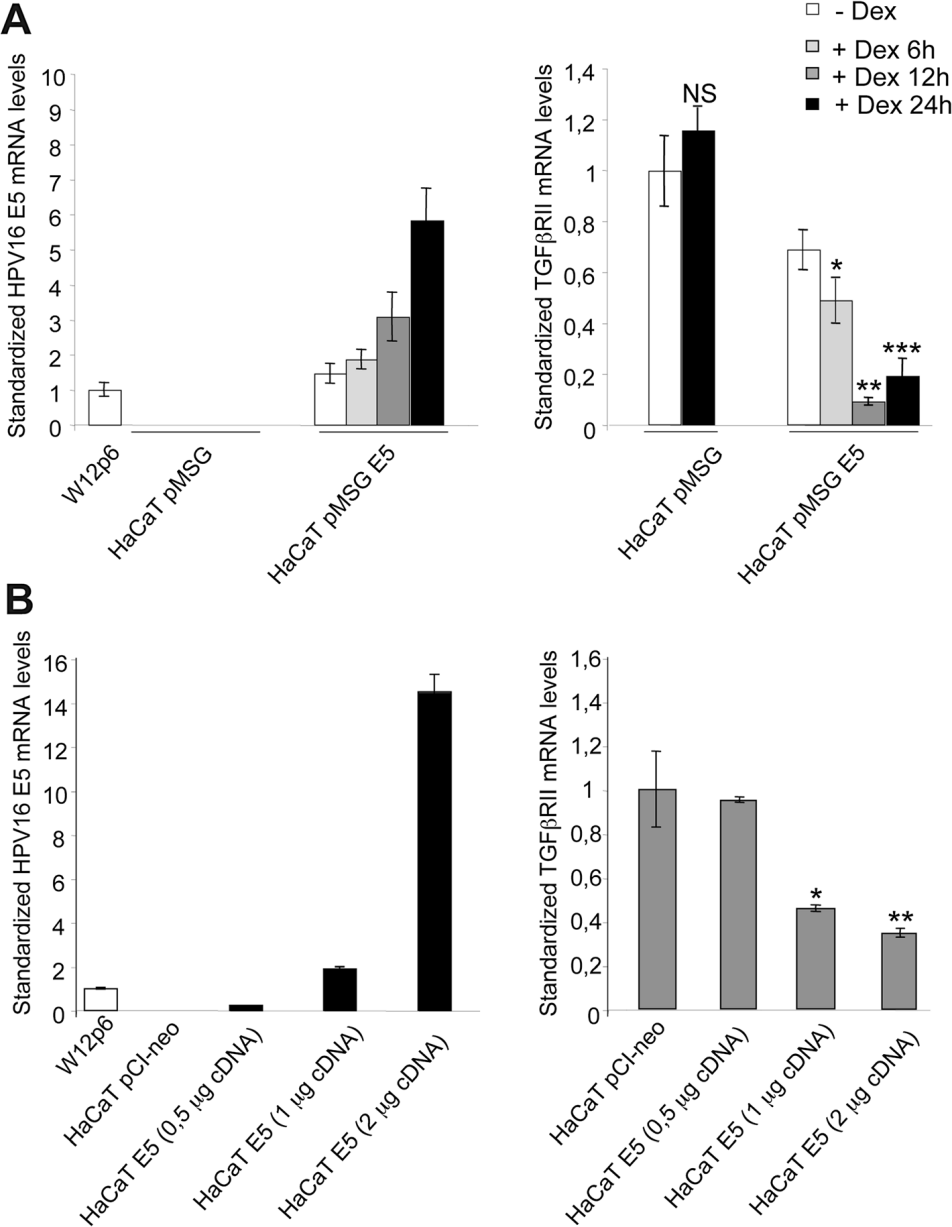
**16E5 is responsible for TGFβRII mRNA down-modulation. A**) HaCaT pMSG E5 were treated with dexamethasone for different times (6 h, 12 h, 24 h). HaCaT pMSG were used as negative control. The 16E5 (left panel) and TGFβRII (right panel) transcript levels were estimated by real-time RT-PCR. Results are expressed as mean values ± standard deviation (SD). Student’s *t* test was performed as reported above NS vs HaCaT pMSG -Dex, *p < 0.001 vs HaCaT pMSG E5 cells -Dex, **p < 0.001 vs HaCaT pMSG E5 cells + Dex 6 h, ***p < 0.05 vs HaCaT pMSG E5 cells + Dex 6 h, **B**) HaCaT cells were transiently transfected with increasing amounts of pCI-neo E5-HA expression vector (0.5 μg, 1 μg and 2 μg) (HaCaT E5) or using the empty vector alone (HaCaT pCI-neo). After transfection, the 16E5 mRNA (left panel) and TGFβRII mRNA (right panel) were quantified by real-time RT-PCR. *p < 0.001 vs HaCaT E5 cells (0.5 μg cDNA), **p < 0.005 vs HaCaT E5 cells (1 μg cDNA).

To evaluate if the expression of 16E5 alone, in the absence of other HPV proteins, could be responsible for the down-modulation of TGFβRII observed *in vivo* in LSILs, we performed a real-time RT-PCR analysis as above using the human keratinocyte cell line HaCaT [21] stably transfected with the construct pMSG 16E5 (HaCaT pMSG E5) [22], in which the expression of the viral protein was progressively induced, by treatment with dexamethasone 1 μM, in a time-dependent manner (6 h, 12 h, 24 h of treatment, Figure 2A, left panel). The HaCaT pMSG cells were used as negative control. 16E5 mRNA levels were normalized with respect to the amount of the viral protein transcript in W12p6. Results are expressed as mean values ± standard deviation (SD). Student’s *t* test was performed as reported in the Additional file 1 Materials and Methods. Results demonstrated that, upon treatment with dexamethasone TGFβRII transcript levels progressively decreased, at least up to 12 h, (Figure 2A, right panel), in cells expressing increasing amounts of 16E5 mRNA (Figure 2A, left panel), suggesting that 16E5 is directly responsible for the receptor transcript down-modulation.

To further demonstrate the role of 16E5 expression in TGFβRII down-regulation, we transiently transfected HaCaT cells using increasing amounts (0.5 μg, 1 μg, 2 μg) of pCI-neo E5-HA expression vector [23] (HaCaT E5), in order to induce an increased expression of 16E5 in a dose-dependent manner (Figure 2B, left panel). HaCaT cells transfected with the empty vector pCI-neo (HaCaT pCI-neo) were used as negative control. Results demonstrated that the TGFβRII transcript levels progressively decreased with the increase of 16E5 mRNA amount (Figure 2A and B, right panels), demonstrating that 16E alone is able to down-modulate the receptor in a dose-dependent and time-dependent manner. Interestingly, this down-modulating effect appears more evident when the viral protein expression is obtained with 1 μg cDNA and is similar to the 16E5 expression in the W12 cell model, representing the most physiological condition.

**Figure 3:**
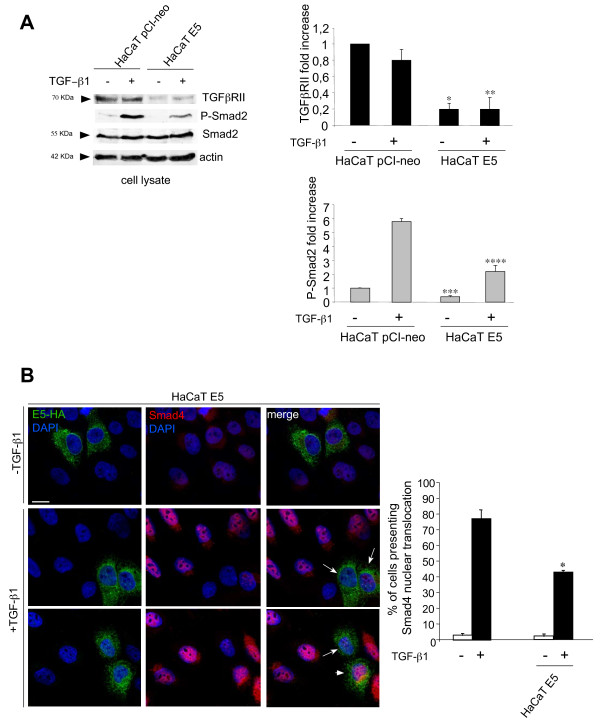
**16E5 expression down-regulates TGFβ1-dependent signaling. A**) HaCaT E5 and HaCaT pCI-neo cells were serum starved for 12 h and then stimulated with 20 ng/ml TGFβ1 for 1 hour at 37°C. Western blot analysis shows reduction of TGFβRII protein and ligand-dependent Smad2 phosphorylation in cells expressing 16E5, while Smad2 protein levels are not affected by 16E5 expression. Densitometric analysis and Student’t test were performed as reported above *, **, ***, **** p < 0.05 vs the corresponding HaCaT pCI-neo cells. **B**) HaCaT E5 cells were stimulated with TGFβ1 as above. Quantitative double immunofluorescence analysis of the Smad4 nuclear translocation upon TFGβ1 treatment in E5-HA positive cells reveals that only a minor portion of the E5 positive cells show Smad4 nuclear translocation (arrowhead), while many of them display Smad4-negative nuclei (arrows). In the quantitative analysis results have been expressed as mean values ± standard errors (SE). p values were calculated using Student’s *t* test.* p < 0.001 vs the surrounding cells. Bar: 10 μm.

To assess whether the down-regulation of TGFβRII expression induced by 16E5 leads to attenuation of TGFβ1 signaling, HaCaT E5 and HaCaT pCI-neo cells were stimulated with 20 ng/ml TGFβ1 for 1 h at 37°C. Western blot analysis confirmed that also the protein expression of TGFβRII is strongly reduced in the presence of E5 (Figure 3A). To analyze the TGFβ/Smad signaling, we first estimated the level of Smad2 phosphorylation in cells stimulated or not with TGFβ1 as above and we found that the ligand-dependent phosphorylation of Smad2 was decreased in cells expressing E5, although the Smad2 total protein amount was not modified (Figure 3A). Densitometric analysis and Student’s test were performed as reported in the Additional file 1 Material and Methods. Since it is well known that, following phosphorylation, Smad2 binds to Smad4 and the Smad2/Smad4 complexes translocate into the nucleus for gene expression regulation [9], we wondered if 16E5 expression could also affect this late event of TGFβ1-mediated signaling. Quantitative double immunofluorescence analysis performed as reported in the Additional file 1 Materials and Methods**,** showed that, among the cells clearly positive for E5 (Figure 3B), the percentage of cells showing Smad4 nuclear translocation upon TGFβ1 treatment (Figure 3B, arrowhead) was significantly decreased compared to the surrounding cells that did not show E5 positivity (Figure 3B). Only a minor fraction of the E5 positive cells showed a Smad4 nuclear translocation (Figure 3B, arrowhead). Thus, taken together our results indicate that 16E5 expression is able to attenuate the TGFβ1/Smad signaling and this loss of signal transduction, leading to destabilization of the epithelial homeostasis [14], may represent an additional mechanism of promotion at very early stages of the HPV-mediated cervical carcinogenesis. Since specific inhibition of the viroporin channel activity of E5 reduces the EGF stimulated mitogenic ERK signaling [24], future work might be addressed to investigate the possible interplay among the TGFβ1/Smad pathway and the EGF/ERK signaling in the presence of 16E5.

## Competing interests

All authors have not financial or non-financial competing interests to declare in relation to this manuscript.

## Authors’ contributions

DF has made substantial contribution to conception and design of the project, she has been involved in drafting the manuscript and has given final approval of the version to be published. FB has made substantial contribution to conception and design of the project, she has been involved in drafting the manuscript and has given final approval of the version to be published. MVM, FM, SR and VF have made substantial contributions to acquisition and analysis of the data and have given final approval of the version to be published. AF has made substantial contributions to acquisition of the data and has given final approval of the version to be published. MRT has made substantial contributions to planning of the entire project, she has been involved in drafting the manuscript and has given final approval of the version to be published. All authors read and approved the final manuscript.

## Supplementary Material

Additional file 1 Materials and Methods. Click here for file
