# Supplementary material for: Expression of HPV16 E5 down-modulates the TGFbeta signaling pathway
Source: Mol Cancer. 2013 May 7;12:38. doi: 10.1186/1476-4598-12-38 (PMC3661392; doi:10.1186/1476-4598-12-38)
Supplement: Additional file 1 — Materials and Methods. [file 1476-4598-12-38-S1.pdf]

Materials and Methods

Etichs statement.

The Institutional Review Board of ‘‘Sapienza’’ University and Sant’Andrea Hospital approved the study protocol (decision n° 176, 03/31/2011) and all enrolled patients gave their written informed consent to the experimental study.

Cytological samples

Cytological samples were obtained from patients attending the Gynaecology Day-Clinic of Sant’Andrea Hospital, Faculty of Medicine and Psychology, “Sapienza” University of Rome. The samples were identified by an alphanumeric code (p1-px, P1-Px, R1-Rx). All samples were HPV16 E6/E7 mRNA positive to NucliSens EasyQ HPV assay (bioMerieux, Marcy l'Etoile, France), which allows the detection of a E6–E7 transcripts in a nucleic acid sequence-based amplification [1], and with a cytology consistent with low grade squamous intra-epithelial lesions (LSIL: n=17) or high grade squamous intra-epithelial lesions (HSIL: n=10) according with the Bethesda classification system 2001. The cytological exam allowed to exclude the presence of contaminating cell types. HPV cervical negative samples (n=4) at the genotyping test (INNOLipa HPV Genotyping kit, Innogenetics, Gent, Belgium) previously described [2] were used as negative controls.

Cells and treatments

The human keratinocyte cell line HaCaT [3] was cultured in Dulbecco’s DMEM, supplemented with 10% fetal bovine serum (FBS) plus antibiotics and were transiently transfected with pCI-neo vector containing 16E5-HA [4] (HaCaT E5) or the pCI-neo empty vector (HaCaT pCI-neo) using jetPEI™ DNA Trasfection Reagent (Polyplus-trasfection, New York, NY, USA) according to manufacturer’s instructions.

HaCaT cells stably transfected with the construct pMSG 16E5 (HaCaT pMSG E5) or with the empty vector (HaCaT pMSG) [5] were cultured in DMEM supplemented with 10% FBS plus antibiotics and were treated with 1 μM dexamethasone to induce 16E5 expression.

The human cervical keratinocyte cell line W12 initiated from a low-grade cervical lesion [6], which retain ~100 to 200 copies of the HPV16 episomes per cell [7; our unpublished data] was cultured as previously described [6] and was used at the passage 6 (W12p6).

Primary culture of normal human ectocervical keratinocytes (HCK) was derived from a patient attending the Gynaecology Unit of Sant’Andrea Hospital who underwent hysterectomy for a clinical reason other than cervical cancer and obtained as previously described [8]. Isolated primary keratinocytes were maintained in Medium 154-CF (Cascade Biologics, Portland, OR, USA)

supplemented with Human Keratinocyte Growth Supplement (HKGS, Cascade Biologics) plus antibiotics and Ca^{2+} 0,03 μM (CascadeBiologics Inc.).

To induce TGFβ signaling, cells were serum starved for 12h and then incubated with 20 ng/ml TGF-β1 (PeproTech, London, UK) for 1h at at 37°C.

Primers

Oligonucleotide primers for HPV16 E5 target gene 5'-CGCTGCTTTTGTCTGTGTCT-3' (sense), 5'-GCGTGCATGTGTATGTATTAAAAA-3' (antisense); and for the β-actin housekeeping gene 5'-CATCAGCAATGCCTCCTGCAC-3' (sense), 5'-GTCATGAGTCCTTCCACGATACCAA-3' (antisense) were chosen with the assistance of the Oligo 5.0 computer program (National Biosciences, Plymouth, MN, USA). Oligonucleotide primers for TGFβRII target gene were previously described [9]. All primers were purchased from Invitrogen (Invitrogen, Carlsbad, CA, USA). Gel electrophoresis was used to verify the specificity of PCR amplicons. For each primer pair, we performed no-template control and no-reverse-transcriptase control (RT negative) assays, which produced negligible signals.

RNA extraction and cDNA synthesis

RNA was extracted using the TRIzol method (Invitrogen) according to manufacturer's instructions and eluted with 0,1% diethylpyrocarbonate (DEPC)-treated water. Each sample was treated with DNAase I (Invitrogen) to avoid any possible DNA contamination. Total RNA concentration was quantitated by spectrophotometry and the quality was assessed by measuring the optical density ratio at 260/280 nm. RNA samples were stored at -80°C. After denaturation in DEPC-treated water at 70°C for 10 minutes, 1 μg of total RNA was used to reverse transcription using iScript™ cDNA synthesis kit (Bio-Rad Laboratoires, Hercules, CA, USA) according to manufacturer's instructions.

PCR amplification and real-time quantitation

Real-time PCR was performed using the iCycler Real-Time Detection System (iQ5, Bio-Rad Laboratories) with optimized PCR conditions. The reaction was carried out in a 96- well plate using iQ SYBR Green Supermix 2X (Bio-Rad Laboratories) adding each forward and reverse primers and 1 μl of diluted template cDNA to a final reaction volume of 15 μl. All assays included a negative control and were replicated three times. The thermal cycling conditions comprised an initial denaturation step at 95°C for 3 minutes, followed by 40 cycles at 95°C for 10 seconds and

55-60°C for 35 seconds. Real-time quantitation was performed with the help of the iCycler IQ optical system software version 3.0a (BioRad Laboratories), according to the manufacturer's manual. The relative expression of the housekeeping gene β-actin was used for standardizing the reaction. The comparative threshold cycle (C_t) method was applied to calculate the fold changes of expression compared to control samples.

Western blot analysis

HaCaT pCI-neo and HaCaT pCI-neo E5 treated with TGF-β1 as above were lysed, 50 μg of total protein were resolved and transferred to reinforced nitrocellulose (BA-S 83, Schleider and Schuell, Keene, NH, USA) as previously described [10]. The membranes were blocked with 5% non fat dry milk in PBS 0.1% Tween 20, and incubated with anti-phospho Smad2 (P-Smad2; Cell Signaling, Denvers, MA, USA), anti-TGFβRII (H567, Santa Cruz Biotechnology Inc., Santa Cruz, CA, USA) monoclonal antibody followed by enhanced chemiluminescence detection (ECL; Amersham, Arlington Heights, IL, USA). The membranes stripped as previously described (Belleudi et al., 2011) and probed again with anti-Smad2 (BD Bioscience, san Jose, CA, USA) monoclonal antibody and anti-actin (Sigma Chemicals, St. Louis, MO., USA) monoclonal antibody, to estimate the equal loading. Densitometric analysis was performed using Quantity One Program (Bio-Rad Laboratoires, Hercules, CA, USA). The resulting values were then normalized and expressed as fold increase respect to the control and mean value ± stardard deviation (SD).

Immunofluorescence

HaCaT E5 grown on coverslips and incubated with TGF-β1 as above were fixed with 4% paraformaldehyde in PBS for 30 minutes at 25°C followed by treatment with 0.1 M glycine for 20 minutes at 25°C and with 0.1% Triton X-100 for additional 5 minutes at 25°C to allow permeabilization. Then cells were incubated with anti-Smad4 (1:10 in PBS; Santa Cruz Biotechnology) polyclonal antibodies and anti-HA (1:50; Covance, Princeton New Jersey, USA) monoclonal antibody. The primary antibodies were visualized with: goat anti-mouse IgG-FITC (1:50 in PBS; Cappel Research Products, Durham, NC, USA) and goat anti-rabbit IgG-Texas red (1:100 in PBS; Jackson Immunoresearch Laboratories, West Grove, PA, USA). Nuclei were stained with DAPI (1:10.000 in PBS; Sigma Chemicals). Cells were scanned in a series of 0.5μm sequential sections with an ApoTome System (Zeiss, Oberkochen, Germany) connected with an Axiovert 200 inverted microscope (Zeiss); image analysis was then performed by the Axiovision software (Zeiss) and 3D reconstruction of a selection of three central out of the total number of the serial optical sections was shown in each figure. Quantitative analysis of the percentage of cells showing Smad4

nuclear translocation was assessed counting for each sample a total of 50 cells, randomly observed in 10 microscopic fields from three different experiments. Results have been expressed as mean values ± standard error (SE).

Statistical Analysis

The HPV 16E5 and TGFβRII mRNA levels from LSIL and HSIL cytological samples were expressed as mean ± 95% confidence interval (CI) from three different experiments in triplicate. To model the relation between the variables, linear regression analysis was used and the Pearson correlation coefficient (r) and 95% CI for r was calculated (CurveExpert data analysis software system).

For real-time PCR, Western blot and Immunofluorescence experiments with transfected cells, p values were calculated using Student's t test and significance level has been defined as $p<0.05$.

References

1. Smits HL, van Gemen B, Schukkink R, van der Velden J, Tjong AHSP, Jebbink MF, ter Schegget J: **Application of the NASBA nucleic acid amplification method for the detection of human papillomavirus type 16 E6-E7 transcripts.** *J Virol Methods* 1995, **54:**75-81.

2. Lorenzon L, Mazzetta F, Venuti A, Frega A, Torrisi MR, French D: **In vivo HPV 16 E5 mRNA: expression pattern in patients with squamous intra-epithelial lesions of the cervix.** *J Clin Virol* 2011, **52:**79-83.

3. Boukamp P, Petrussevska RT, Breitkreutz D, Hornung J, Markham A, Fusenig NE: **Normal keratinization in a spontaneously immortalized aneuploid human keratinocyte cell line.** *J Cell Biol* 1988, **106:**761-771.

4. Ashrafi GH, Haghshenas MR, Marchetti B, O'Brien PM, Campo MS: **E5 protein of human papillomavirus type 16 selectively downregulates surface HLA class I.** *Int J Cancer* 2005, **113:**276-283.

5. Oelze I, Kartenbeck J, Crusius K, Alonso A: **Human papillomavirus type 16 E5 protein affects cell-cell communication in an epithelial cell line.** *J Virol* 1995, **69:**4489-4494.

6. Stanley MA, Browne HM, Appleby M, Minson AC: **Properties of a non-tumorigenic human cervical keratinocyte cell line.** *Int J Cancer* 1989, **43:**672-676.

7. Pett MR, Alazawi WO, Roberts I, Dowen S, Smith DI, Stanley MA, Coleman N: **Acquisition of high-level chromosomal instability is associated with integration of human papillomavirus type 16 in cervical keratinocytes.** *Cancer Res* 2004, **64:**1359-1368.

8. Cardinali G, Ceccarelli S, Kovacs D, Aspite N, Lotti LV, Torrisi MR, Picardo M: **Keratinocyte growth factor promotes melanosome transfer to keratinocytes.** *J Invest Dermatol* 2005, **125:**1190-1199.

9. Soufla G, Sifakis S, Baritaki S, Zafiropoulos A, Koumantakis E, Spandidos DA: **VEGF, FGF2, TGFB1 and TGFBR1 mRNA expression levels correlate with the malignant transformation of the uterine cervix.** *Cancer Lett* 2005, **221:**105-118.

10. Belleudi F, Leone L, Purpura V, Cannella F, Scrofani C, Torrisi MR: **HPV16 E5 affects the KGFR/FGFR2b-mediated epithelial growth through alteration of the receptor expression, signaling and endocytic traffic.** *Oncogene* 2011, **30:**4963-4976.
